# Upper-body isometric horizontal strength in game sport athletes

**DOI:** 10.3389/fspor.2023.1213957

**Published:** 2023-06-16

**Authors:** Lukas Reichert, Till Müller, Björn Wieland, Marie-Therese Fleddermann, Karen Zentgraf

**Affiliations:** Work Unit Movement and Exercise Science in Sports, Institute of Sport Sciences, Goethe University Frankfurt, Frankfurt am Main, Germany

**Keywords:** sports performance, professional athletes, expertise, side-to-side differences, validation

## Abstract

Imagine blocking the opposing defense linemen in American football to protect your quarterback or creating gaps in the opponents' defense by setting blocks as a pivot player in handball. Such movements require a pushing action away from the body with the arms and stabilizing the whole body in different postural positions. Upper-body strength obviously plays an important role during American football and handball as well as in other game sports with opponent contact such as basketball. Yet, the availability of appropriate tests to measure upper-body strength serving sport-specific requirements seems limited. Therefore, a whole-body setup to measure isometric horizontal strength in game sport athletes was developed. The purpose of the study was to verify validity and reliability of this setup and present empirical data from game sport athletes. In 119 athletes, isometric horizontal strength was measured in three game-like standing positions (upright, slightly leaning forward and clearly leaning forward), each in three weight-shift conditions (80% of body weight on the left leg, 50/50% on both legs, 80% on the right leg). Also, handgrip strength on both sides was measured in all athletes using a dynamometer. Linear regression indicated that handgrip strength is a significant predictor of upper-body horizontal strength in female (*β* = 0.70, *p* = 0.043) but not in male athletes (*β* = 0.31, *p* = 0.117). As an expertise-related factor, linear regression indicated that the number of years played at the top level is a predictor of the upper-body horizontal relative strength measure (*β* = 0.05, *p* = 0.03). Reliability analyses showed high levels of within-test reliability (ICC > 0.90) as well as test-retest reliability between two separate measurements (*r* > 0.77). The results indicate that the setup used in this study could be a valid tool for measuring performance-relevant upper-body horizontal strength in different game-like positions in professional game sport athletes.

## 1. Introduction

Besides technical or tactical demands, professional game sport athletes (GSA) need to exhibit an adequate athletic performance to be successful in their sports. Previous studies have highlighted the importance of lower body neuromuscular strength for athletic performance. Thus, generating maximum strength and a high rate of force development is required during high-intensity actions such as jumping, sprinting, agility, and change of direction (1–3). However, in game sports such as American football, basketball, handball, but also volleyball or tennis, many sport-specific actions such as serving, throwing, hitting, spiking, blocking, and tackling require upper-body force production when athletes have different standing positions. In American football, for example, opponents must be blocked to give the quarterback enough time to make the play, or in handball, the attackers must be prevented from throwing and must be defended (pushed) away from the goal to do so. From this perspective, upper-body horizontal strength (UBHS), e.g., the ability to generate pushing strength into a horizontal direction, seems to play a crucial role in game sports performance (1, 4, 5). Up to now, only a few studies have investigated UBHS as a performance-determining factor. In volleyball, for example, Gonçalves et al. (6) postulated that upper-body power measured by medicine ball throw can be a discriminating factor between elite and sub-elite players. In addition, Milić et al. (7) reported that more successful young female volleyball players exhibited better upper-body power (as the velocity-oriented aspect of strength) than less successful players. Furthermore, in sports that involve contact and collisions during play, such as in rugby or American football, studies suggest that UBHS measured by one-repetition-maximum bench press contributes to tackling performance with small to moderate differences between players' levels (2, 8). Players with higher UBHS values were more likely to be selected for the team (9) and to have reduced injury risk when tackling (2). In female rugby sevens, the players with greater levels of UBHS were those who had more playing minutes in comparison to other teammates, which underlines that UBHS is a performance-determining factor in game sports (10).

Additionally, GSA must be able to generate UBHS in different sport-specific positions and, accordingly, perform actions not only in symmetric lower extremity positions, but often shifted to one leg side or in a more upright or bent forward position. American football and rugby players, for example, tackle their opponents not only frontally but also on the left or right side depending on the opponents' route and their changes of direction (11–13). In basketball, players on offense set blocks in different (laterally shifted) positions in order to run plays (e.g., pick and roll). As described above, in handball, defenders prevent attackers from throwing by pushing them horizontally away from the goal. Another example in handball is the pivot player. His job is to set blocks in different positions, thus creating gaps in the opponents' defense. Therefore, the pivot player usually has to physically push against one or more defenders, thus UBHS in laterally-shifted positions is required in order to attack the goal. It seems plausible that side-to-side differences (DIFF) in UBHS may also lead to different performance in game actions. While DIFF and their relationship to sports performance have especially been investigated in lower extremities (e.g., in sprinting, jumping or change of direction; 14, 15), only a few studies have investigated DIFF for upper-body performance. Zemkova et al. (16) reported DIFF as peak and mean values of power (14%–18%) during trunk rotations between the dominant and non-dominant sides of ice-hockey, tennis players and golfers. Moreover, they postulated that side-related imbalances might result in reduced game performance. Furthermore, Chapelle et al. (17) found DIFF in both upper- and lower-body tests. In addition, previous studies have shown that both UBHS strength values in American football youth athletes (18) as well as DIFF in lower extremities vary throughout the season in professional cricket athletes (19). Therefore, such aspects should be monitored on a regular basis. Nonetheless, upper-body tests in which DIFF can be detected are rarely performed.

According to previous studies, maximum strength is often tested isometrically. An isometric strength test that is often performed for measuring upper-body strength in GSA is the handgrip strength (GS) test (20–23). GS is shown to be a predictor of overall muscle strength across ages (20) as well as a performance-relevant parameter in sports in which, for example, throwing a ball (e.g., handball or basketball 22, 24); or swinging a bat (e.g., ice-hockey or tennis; 23, 25) plays an important role for game performance. For example, in ice-hockey players, Peterson et al. (25) could show that Division I ice-hockey players exhibited significantly higher GS values compared to Division III players. In addition, increased GS is associated with greater upper-body strength (21). Further studies also used isometric strength tests such as the maximum bench press (8, 26) for measuring upper-body strength. Studies also quantified the power component of the upper-body by a bench press throw or medicine ball throw (1, 5, 6, 26, 27). However, upper-body movements, such as generating strength in a horizontal direction, are often the result of a whole-body movement standing in an upright position with multiple segments working together in a kinetic chain. For example, when blocking an opponent, strength is generated through the lower-body (feet and legs) and is transferred through the core (abdominal/back muscles) to the upper-body (chest and upper extremities). This type of movement pattern is mostly neglected in tests such as mentioned above. Despite the reported impact of UBHS on performance, the availability of appropriate tests to measure UBHS levels in a game-sports relevant manner seems limited (8, 28). Therefore, it seems necessary to examine methodologies to capture such game-specific requirements.

In summary, present measurements focus more on isolated and bilateral procedures. There is less information on UBHS measured in game-like standing positions considering DIFF, which are common in game sports. Therefore, the purpose of this study was to investigate UBHS in a game-like standing position in a whole-body oriented setup. Based on this, the aim was to evaluate validity and reliability of our setup as well as to examine strength values of GSA in all positions, to verify whether this setup may be used for further diagnostics to evaluate game-relevant UBHS performance.

## 2. Materials and methods

The present study was conducted in a cross-sectional design to determine whether a whole-body oriented setup would be a valid tool for measuring isometric UBHS in GSA. All tests were performed between March 2021 and February 2022. Prior to testing, participants received detailed written and verbal information about the possible benefits and risks associated with this study. Written informed consent was obtained from the participants (as well as from parents if athletes were under 18 years old). The study was approved by the local ethics committee (Chair: Klein, A.; 2021–30, June 28th, 2021).

### 2.1. Participants

One hundred and nineteen professional GSA (volleyball *n* = 18 male, *n* = 18 female; 3 × 3 basketball *n* = 17 male, *n* = 12 female; ice-hockey *n* = 19 female; 5 × 5 basketball *n* = 21 male; handball *n* = 11 male; American football *n* = 2 male; soccer *n* = 1 female) participated in this study (age = 22.70 ± 5.21 years). All GSA were acquired via cooperation contracts with national and regional clubs as part of a project which was supported by the German Federal Institute of Sports Science (BISp) between 2021 and 2022 (title: “Motor profiles in game sports”, grant number 070503/21–22). GSA were included if they practiced their sport professionally (practiced twice a day and/or earned their living from their sport) and/or were currently part of a squad (youth or national). Subjects were excluded if they were injured at the time of testing or shortly before. Further personal information, such as the number of years that the GSA played at the top level were assessed as expertise-related parameters via self-report. Prior to measurement, a 20-minute standardized warm-up program consisting of running, mobilization of major joints, dynamic stabilization and coordination tasks was performed. The warm-up program was supervised and standardized by a professional strength and athletics coach. Following warm-up, GSA were instructed for the measurement. In terms of standardization, the same two persons instructed the athletes and collected data for this study. A test manual was prepared in advance to ensure standardized instructions.

### 2.2. Handgrip strength (GS)

GS was measured unimanually using a hand-held dynamometer (MicroFET2; Hoggan Scientific, Salt Lake City, USA). GSA were asked to sit on a chair at a table. The sitting position was adjusted so that both feet were placed on the floor with a 90° knee angle; forearms were on the table with 90° elbow flexion. The wrist joint on the measured side had to be in an extended position. The other arm had to rest loosely on the table. GSA were instructed to press lightly at the beginning of the measurement and to increase their force during a 3-second countdown so that they were at their maximum force when 0 was reached. They were asked to continue pressing maximally for another 5–6 s until the test administrator announced a stop. Before the actual measurement, GSA received one test trial for each side. Two trials were performed for each side. If GSA failed to perform the measurement correctly, a third trial was performed. The maximum strength value in Newton for each side was documented. For further analysis, also relative strength values were calculated by dividing the absolute strength value by the athletes' body weight (N/kg).

### 2.3. Isometric upper-body horizontal strength (UBHS)

**Figure 1:**
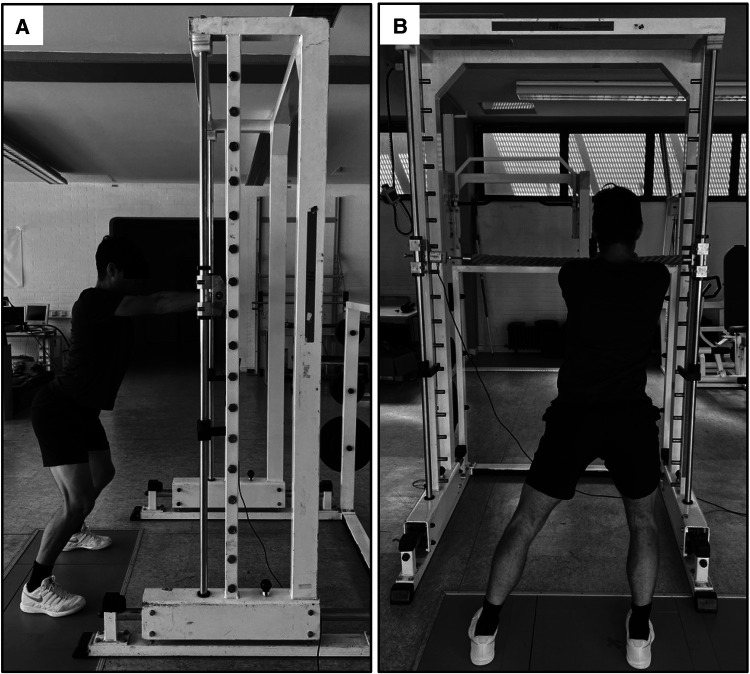
UBHS measured in a whole-body oriented setup in the slightly leaned forward position (position 2) in the 50/50% (**A**) and 80% on the right leg condition (**B**).

**Figure 2:**
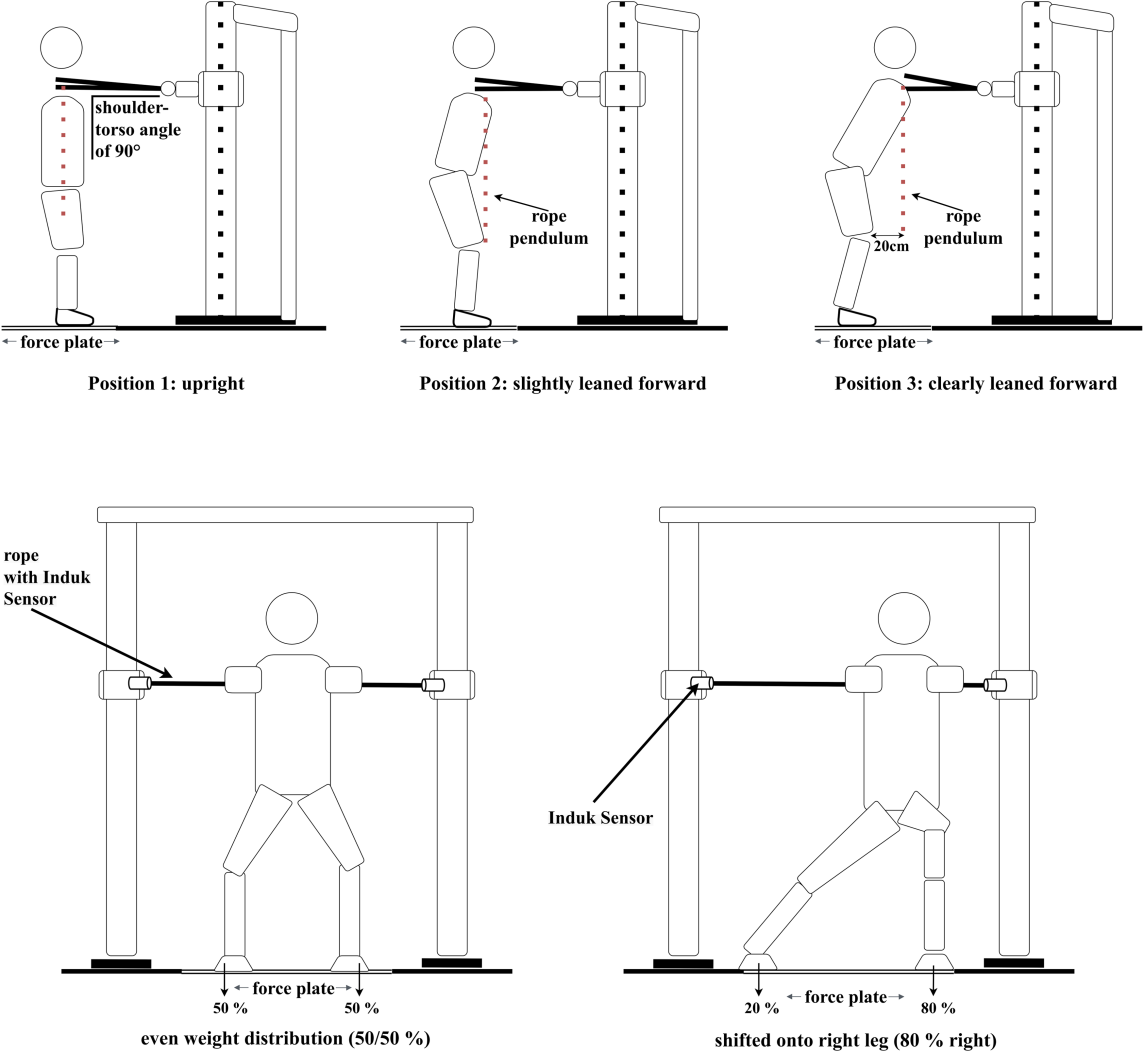
Self-developed, whole-body oriented setup in which the isometric upper-body horizontal pushing strength was measured in a standing position. UBHS was conducted in three different positions (position 1: upright, position 2: slightly leaned forward, position 3: clearly leaned forward), each in three different weight-shift conditions during pushing [80% on the left leg (not shown in the Figure), 50/50% and 80% on the right leg]. The positions were controlled via a rope pendulum and the weight-shift conditions were monitored via a floor-embedded force plate.

UBHS was measured using a self-developed, whole-body oriented setup with an Induk electrical force transducer installed on a rope (Type 761, Induk, Wuppertal, Germany, diameter: 4.5 cm). The construction of the apparatus and the positions employed are shown in Figures 1, 2. The aim of the measurement was to push maximally against the rope into a horizontal direction in various postural positions. To cover different game-sport positions, measurements were performed in three different positions: upright (position 1), slightly leaned forward (position 2) and clearly leaned forward (position 3), each with three different weight distributions during pushing (80% on the left leg, 50/50%, 80% right leg), thus, in total, nine conditions.

To generate individual values for the weight-shifted conditions, we asked GSA to stand still on a floor-embedded piezoelectric force plate (40 cm × 60 cm, Type 9,851, Kistler, Winterthur, Switzerland); to calculate 20%, 50% and 80% of weight force respectively [e.g., with a value of 1,000 N, they had to lean with 800 N (80%) onto their right leg in the right-shifted condition; see Figure 2].

For the measurement, GSA were asked to stand with their feet shoulder-width apart and with knees slightly bent in a self-chosen game-like power-position while grabbing a rope in front of them (see Figure 2). To standardize this position and to ensure that GSA are taking up this position for each measurement, the distance from the floor to the iliac crest (ranging from 95 cm to 110 cm, depending on individual anthropometrics) was measured at the beginning of the measurement and before each single trial. The distance between each individual GSA and the rope was controlled using a rope pendulum which was positioned at the shoulder level. Starting in their game-like position, GSA were asked to grab the rope with outstretched arms, elbows locked, in front of their body with their hands touching each other (see Figure 2). In position 1, the distance between GSA and rope was adjusted to ensure that they were standing in an upright position (shoulder over hip) and having a shoulder-torso angle of 90°. The height of the rope was kept constant (according to position 1) in all three positions. However, the distance between rope and GSA was adapted for positions 2 and 3 (see Figure 2). In the second position, GSA stood slightly leaned forward (shoulder over knee), while in the third position they were clearly leaned forward (shoulder 20 cm in front of the knee). By allowing GSA to choose their own game-like power position, joint angles were adapted on an individual basis, with knee angles ranging from 110° to 140° and hip angles ranging from 110° to 160° over all GSA depending on their individual anthropometrics. Depending on the position, joint angles in hip and knee were larger in the more upright position (position 1; e.g., 160° hip angle and 140° knee angle), smaller in the slightly leaned forward position (position 2; e.g., 150° hip angle and 135° knee angle) and the smallest in the clearly leaned forward position (position 3; e.g., around 140° hip angle and 130° knee angle).

Measurements were performed starting in position 1 due to calibration requirements, followed by position 2 and position 3. In each of the three positions, GSA started in an evenly distributed position (50/50%) and then took up weight-shifted positions in a permuted order. During the measurement, GSA had to push against the rope, increasing their strength during a 3-second countdown, and pressing maximally for another 6 s. To ensure horizontal pushing, GSA were instructed to keep their gaze between their hands during the measurement. For weight-shifted positions, GSA were asked to push maximally when standing with 80% of their weight shifted onto their left leg (80% left), or when shifted 80% of their weight onto their right leg (80% right), and to maintain these shifted positions during pushing. The weight-shifted positions were monitored via the floor-embedded force plate. Feedback on execution of the task was provided between trials. Two trials were performed for each condition. If the athletes failed to perform the measurement correctly (e.g., leaving their position prematurely and/or shifting their weight by more than 10%), a third trial was performed.

The maximum value of each measurement was generated via MatLab (R2020b) using a moving mean function over 0.5 s (29). In order to eliminate possible initial strength peak values (e.g., in case GSA let themselves fall towards the rope and, thereby, generate an initial peak which does not represent a valid strength measure), the moving mean was calculated only from the third second onwards. The maximum moving mean value for each measurement in the respective position was taken. For further analysis, relative strength values (N/kg) were calculated by dividing the absolute UBHS (*N*) value by the GSA's body weight (kg).

### 2.4. Statistical analysis

The data are presented as means (M) and standard deviations (SD). All statistical analyses were performed using IBM SPSS version 26 for Macintosh (IBM, New York, USA). For reliability analysis, we calculated intra-class correlation coefficients (ICC). Interpretation of ICC values was in accordance with Koo & Li (30), with values >0.90 = excellent, >0.75–0.90 = good, 0.50–0.75 = moderate, and <0.50 = poor. Even though the project in which the study was conducted was designed as a cross-sectional study, we were able to recruit *n* = 10 athletes for a second measurement at an interval of 4–6 months. We used those data to check for test-retest reliability. Since we found high ICC values for position 2 as well as to economize the testing, we decided to measure only position 2 in a retest. For validation, a linear regression analysis was calculated to investigate the relationship between UBHS within this setup and GS as an indicator for overall muscle strength. Therefore, mean relative UBHS values over all nine positions as well as relative GS values over both hands were calculated. Based on the knowledge of the correlation between GS and overall muscle strength, we hypothesized that GS explains some variance of UBHS. Furthermore, to check for the plausibility in terms of performance, the relationship between the number of years played at the top level (as the expertise-related factor) and strength values was verified using a linear regression analysis. Mean relative strength values for each position were calculated and used as dependent variable. We hypothesized that athletes with higher expertise would exhibit higher relative strength values. To investigate how UBHS values are affected by the different leaned-forward positions and whether athletes show side-to-side differences between the weight-shifted positions (left, 50/50%, right), a 3 × 3 (position, shift) repeated-measures ANOVA was conducted for both the male and female groups. Normality assumptions were verified by using the Shapiro–Wilk test (*p *> 0.05). The level of statistical significance was set at *α* < 0.05. Effect sizes were interpreted according to Cohen (31): <0.06 = small effect, 0.06–0.14 = moderate effect and >0.14 = large effect.

## 3. Results

The means and standard deviations for each position in UBHS as well as for the left and right hand in GS are shown in Table 1.

**Table 1:** Mean ± SD in N/kg for each position of UBHS as well as for left and right hand in GS.

	UBHS - Position 1			UBHS - Position 2			UBHS - Position 3			GS	
	Shifted onto left leg	Even 50/50% both legs	Shifted onto right leg	Shifted onto left leg	Even 50/50% both legs	Shifted onto right leg	Shifted onto left leg	Even 50/50% both legs	Shifted onto right leg	Left Hand	Right Hand
Total (*n* = 119)	3.06 ± 0.96	3.33 ± 0.91	3.07 ± 0.93	4.43 ± 1.07	4.81 ± 1.18	4.24 ± 1.14	5.94 ± 1.53	6.24 ± 1.44	5.65 ± 1.47	3.18 ± 0.54	3.40 ± 0.59
Male (*n* = 69)	2.89 ± 0.90	3.23 ± 0.83	2.92 ± 0.86	4.23 ± 0.99	4.61 ± 1.01	4.01 ± 0.98	5.51 ± 1.36	6.05 ± 1.32	5.25 ± 1.27	3.29 ± 0.56	3.52 ± 0.63
Female (*n* = 50)	3.29 ± 1.00	3.47 ± 1.00	3.28 ± 0.99	4.70 ± 1.14	5.10 ± 1.34	4.57 ± 1.27	6.55 ± 1.56	6.52 ± 1.57	6.20 ± 1.56	3.04 ± 0.49	3.23 ± 0.49

### 3.1. Reliability of UBHS

ICC values and test-retest correlation coefficients are shown in Table 2. All positions show excellent ICC levels with values >.90 (30). Position 2 shows high levels of test-retest reliability with *r* = 0.77–0.80 (31).

**Table 2:** Intra-class correlation coefficients between the first and second trial in each position and test-retest reliability for position 2 of UBHS.

UBHS (*n* = 119)	Intra-class correlation (*n* = 119)	Test-retest reliability (*n* = 10)	
		r	p
**Position 1**			
Shifted onto left leg	0.94	/	/
Even 50/50% both legs	0.91	/	/
Shifted onto right leg	0.94	/	/
**Position 2**			
Shifted onto left leg	0.96	0.80	0.006
Even 50/50% both legs	0.93	0.78	0.008
Shifted onto right leg	0.97	0.77	0.010
**Position 3**			
Shifted onto left leg	0.96	/	/
Even 50/50% both legs	0.97	/	/
Shifted onto right leg	0.97	/	/

### 3.2. Validity of UBHS

**Figure 3:**
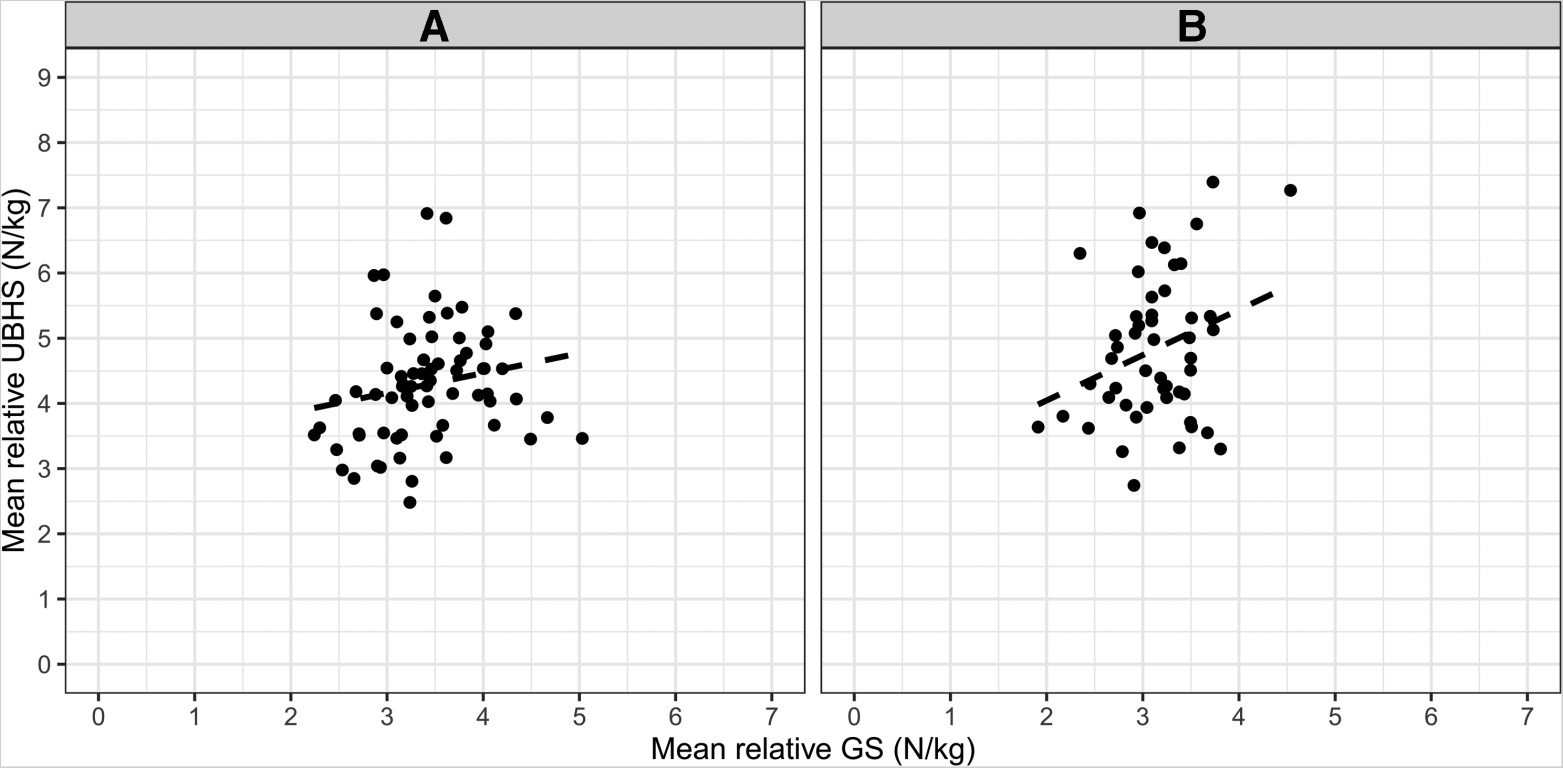
Scatterplot of mean relative UBHS values (N/kg) and mean relative GS values (N/kg) each for male (**A** – left side; *n* = 69) and female (**B** – right side; *n* = 50) GSA.

For validation, a linear regression analysis was conducted to investigate the relationship between mean relative UBHS values over all nine positions within the setup used in this study and mean relative GS values of both hands as an indicator for overall muscle strength. The scatterplots are shown in Figure 3. Assumptions for performing linear regression were checked by visual inspection (linearity, normality and homoscedasticity). Autocorrelation of residuals was verified using the Durbin–Watson statistic (male = 1.92, female = 2.19). For the male (*R^2^* = 0.04, adjusted *R^2^* = 0.02) and female GSA (*R^2^* = 0.08, adjusted *R^2^* = 0.06) the overall model indicates a small goodness-of-fit according to Cohen (31). The mean relative GS values can statistically significantly predict mean relative UBHS values in female GSA [*F*(1, 48) = 4.34, *β* = 0.70, *p* = 0.043] but not for male GSA [*F*(1, 66) = 2.52, *β* = 0.31, *p* = 0.117].

For establishing plausibility of measurement in terms of game performance, i.e., to investigate the relationship between an expertise-related factor and UBHS and to clarify whether athletes with higher expertise exhibited higher UBHS values in our setup, we conducted a linear regression analysis between the number of years played at the top level (4.35 ± 4.80 years) and the mean UBHS values in position 2. Assumptions for performing linear regression were checked by visual inspection (linearity, normality and homoscedasticity). Autocorrelation of residuals was verified using the Durbin–Watson statistic (Durbin–Watson = 1.94). The overall model (*R^2^* = 0.04, adjusted *R^2^* = 0.04) indicates a small goodness-of-fit according to Cohen (31). The number of years played at the top level of sport significantly predict UBHS, *F*(1, 116) = 5.28, *β* = 0.05, *p* = 0.02. Similar results were obtained for position 3 [*F*(1, 116) = 6.41, *p* = 0.01, adjusted *R*^2^ = 0.04] but not for position 1 [*F*(1, 117) = 3.10, *p* = 0.08].

#### 3.3. Factorial ANOVA

Mauchly's test of sphericity indicated that the assumption of sphericity was met for the male (*p* = 0.159) but not for the female GSA (*p* = 0.048). Therefore, Greenhouse–Geisser adjustment was used to correct for violations of sphericity in the female data set. There was a significant interaction effect for positions and shifts for the male GSA, *F*(4, 272) = 8.93, *p *< 0.001, partial *η*^2^ = 0.12, as well as for the female GSA, *F*(3.47, 166.36) = 3.86, *p* = 0.005, partial *η*^2^ = 0.07. This indicates that strength values were affected differently by positions. Over all positions, male GSA generated the highest strength values when standing in the even 50/50% weight distribution (1_50_ = 3.23 ± 0.83 N/kg, 2_50 _= 4.61 ± 1.01 N/kg, 3_50_ = 6.05 ± 1.32 N/kg). In position 1, male GSA exhibited similar strength values when their weight was shifted onto the right leg (1_right _= 2.92 ± 0.86 N/kg) and the left leg during pushing (1_left _= 2.89 ± 0.90 N/kg). However, in positions 2 and 3, they showed slightly higher strength values when their weight was shifted onto the left leg (2_left _= 4.23 ± 0.99 N/kg; 3_left _= 5.51 ± 1.36 N/kg) compared to the right leg during pushing (2_right _= 4.01 ± 0.98 N/kg; 3_right _= 5.25 ± 1.27 N/kg). The Bonferroni-adjusted post-hoc tests revealed a significant difference between the 50/50% weight distribution and a shift onto the left and right leg in all positions (*p* < 0.001) and a significant difference between the shift onto the left and right leg in position 2 (*p* = 0.012) and in position 3 (*p* = 0.004) but not in position 1 (*p* > 0.05).

Female GSA showed similar relative strength values in the first (upright) position compared to the male GSA (1_left _= 3.28 ± 1.01 N/kg; 1_50 _= 3.45 ± 1.01 N/kg; 1_right _= 3.26 ± 0.98 N/kg). However, in a clearly leaned forward position, female GSA showed similar values when weight was shifted onto the left leg and in the evenly distributed position during pushing (3_left _= 6.55 ± 1.56 N/kg; 3_50 _= 6.52 ± 1.57 N/kg; 3_right _= 6.20 ± 1.56 N/kg). Bonferroni-adjusted post-hoc tests did not show significant differences between the weight shifts in position 1 (*p* > 0.05). However, significant differences were found between the 50/50% distribution and a shift onto the left (*p* = 0.001) and right leg (*p* < 0.001) in position 2 and a weaker, but still significant, difference between the 50/50% distribution and a shift onto the right leg (*p* = 0.011) in position 3.

Regarding positions, Mauchly's test of sphericity indicated that the assumption of sphericity was not met for male and female GSA (*p *< 0.05). The Greenhouse–Geisser adjustment was used to correct for the violation of sphericity. There was a main effect for “position” for the male, *F*(1.53, 103.67) = 412.05, *p *< 0.001, partial *η*^2^ = 0.86, and female GSA, *F*(1.46, 70.25) = 325.79, *p *< 0.001, partial *η*^2^ = 0.87. Bonferroni-adjusted post-hoc analysis revealed significant differences (*p *< 0.001) between all positions for the male and female GSA. Male and female GSA showed higher strength values in position 3 (3_male _= 5.60 ± 0.15 N/kg; 3_female _= 6.43 ± 0.21 N/kg) than in position 2 (2_male_ = 4.28 ± 0.11 N/kg; 2_female_ = 4.79 ± 0.17 N/kg) and position 1 (1_male_ = 3.01 ± 0.10 N/kg; 1_female_ = 3.33 ± 0.13 N/kg).

For body-shifted positions, the assumption of sphericity was met for both male and female GSA (*p *> 0.05). The analyses showed a significant main effect for weight-shifted positions in the male, *F*(2, 136) = 56.43, *p *< 0.001, partial *η*^2^ = 0.45 and female GSA, *F*(2, 96) = 10.69, *p *< 0.001, partial *η*^2^ = 0.18. The Bonferroni-adjusted post-hoc tests showed significantly higher strength values for the male GSA in a 50/50% weight distribution (50/50 = 4.63 ± 0.11 N/kg) compared to a shift onto the left (left = 4.21 ± 0.12 N/kg, *p *< 0.001) and the right leg during pushing (right = 4.06 ± 0.11 N/kg, *p *< 0.001) and between a shift onto the left and right leg (*p* = 0.03). For female GSA, Bonferroni-adjusted post-hoc tests showed significantly higher values in the 50/50% distribution (50/50 = 5.02 ± 0.17 N/kg) compared to a shift onto the left (left = 4.84 ± 0.16 N/kg, *p* = 0.04) and onto the right leg (right = 4.68 ± 0.17 N/kg, *p *< 0.001), but not between a shift onto the left or right leg during pushing (*p *= 0.10).

## 4. Discussion

In this study, we have measured UBHS of professional male and female GSA in game-like standing laterally shifted positions. The aim of our study was to determine validity and reliability of our measurements. Both criteria can be assessed using various parameters (32). In order to verify within-test reliability, we calculated ICC values. All positions showed excellent ICC values with values >.90, indicating high levels of within-test reliability. In addition, to verify test-retest reliability, we measured 10 athletes in a retest. Measuring all nine conditions was very time-consuming. Thus, since high reliability for position 2 was noted, we decided to measure only position 2 in a retest. Results showed a high correlation (*r* > 0.77) between both measurements. Comparing reliability of our setup to that of other studies or devices, we found similarly high reliabilities (28, 33). As our test-retest analysis only comprised *n* = 10, results should be interpreted cautiously. Nevertheless, analysis indicates that re-measurements of position 2 are reliable.

For validation, we conducted a linear regression analysis between mean relative UBHS and mean relative GS values. In previous studies, GS is measured in an isometric setup which is often used as an indicator for general muscle strength as well as sport expertise levels (20, 21). As GS is a predictor of overall muscle strength (20), we hypothesized that GS explains a substantial amount of variance in UBHS. The results indicated that GS values predict UBHS values for female (adjusted *R^2^* = 0.06) but not for male athletes (adjusted *R^2^* = 0.02). Looking at the GS values, male GSA exhibited greater GS values than female GSA which is consistent with previous findings (34). Considering the relationship between GS and UBHS, the small effects in males may be in part attributed to sports specificity. Regarding the relevance of GS in basketball, studies provide inconsistent findings. For example, Ramos et al. (22) found that GS, combined with stature, agility and countermovement jump, is a good predictor for game performance in young male basketball players. However, in contrast, McGill et al. (35) demonstrated a negative relationship between GS and performance-relevant parameters such as steals, rebounds and minutes played in male university basketball athletes. Accordingly, the performance-relevant role of GS in basketball does not seem to be fully clarified. In accordance with this, male basketball athletes had lowest GS values (3.30 ± 0.54 N/kg) with rather higher UBHS values (4.51 ± 1.20 N/kg) compared to other male GSA in this sample (GS = 3.45 ± 0.58 N/kg, UBHS = 4.21 ± 0.75 N/kg). Since basketball players (*n* = 21) were slightly overrepresented in the male sample, this might be a possible explanation for the small effects. As shown above, male basketball players exhibited rather high variance in UBHS compared to other male GSA, which could further amplify this small effect. Therefore, this hypothesis can only be confirmed to a limited extent. In addition, in contrast to previous studies reporting strong correlations for new measurement devices (28), the rather small correlation found in this study could be due to different measurement setups. Whereas GS measurements capture underarm and hand muscle strength, UBHS related to activation of multiple muscle groups over the whole body. Results indicate that GS explains some variance in UBHS - but only to a limited extend. Accordingly, measuring UBHS in this setup gives additional insights into performance-relevant parameters in GSA.

**Figure 4:**
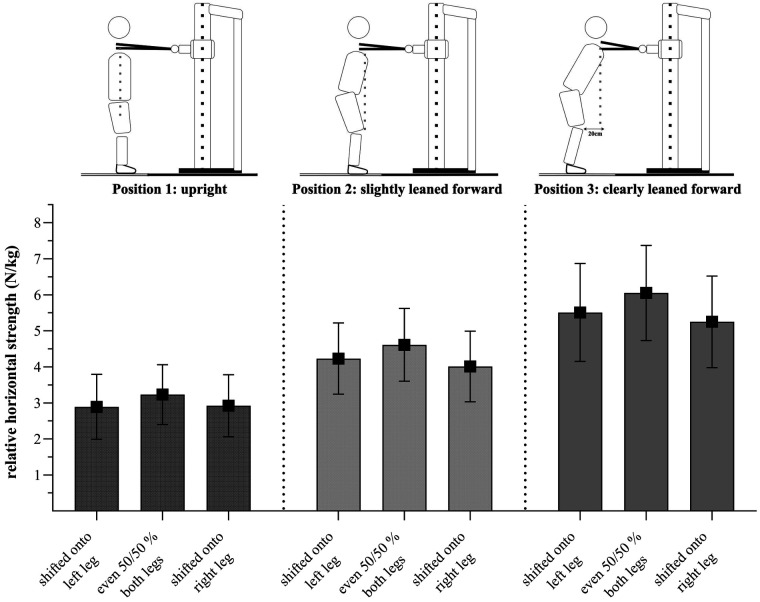
Relative strength values (N/kg) presented as mean ± standard deviation for each position/weight shift for male GSA (*n* = 69).

**Figure 5:**
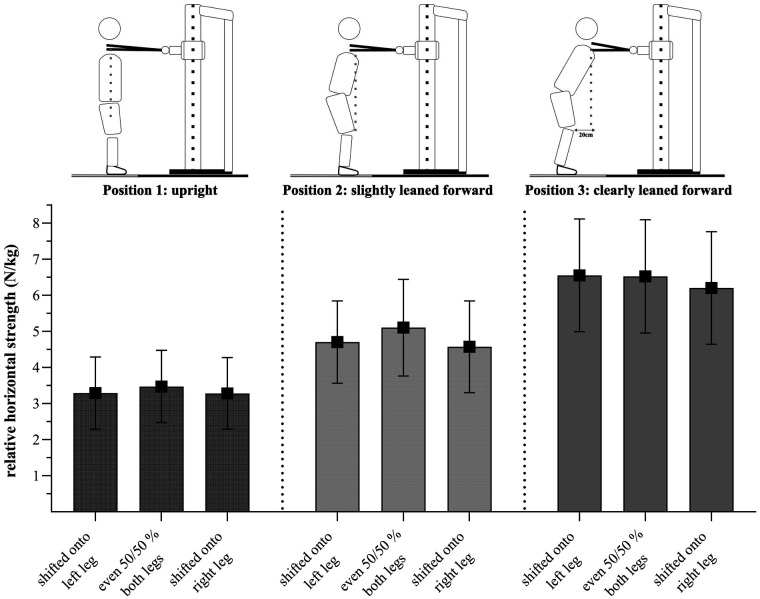
Relative strength values (N/kg) presented as mean ± standard deviation for each position/weight shift for female GSA (*n* = 50).

For establishing plausibility of measurements, we conducted a linear regression analysis between the number of years played at the top level and the mean UBHS values in position 2 hypothesizing that GSA with higher expertise exhibit higher UBHS values compared to GSA with less expertise. However, our sample was quite heterogeneous in terms of expertise between genders. Our sample included male GSA with rather low expertise (2.51 ± 3.20 years played on top level) and older female GSA with rather high expertise (6.89 ± 5.47 years played on top level). Thus, the number of years played at top level seems to be confounded by gender. According to Bartolomei et al. (36), male GSA show superior performance in upper- and lower-body tests compared to female GSA. With this in mind, we assume that such differences between male and female GSA would also be reflected in upper-body horizontal strength measures, with higher values in UBHS for males. Given this expectation and the fact that our sample shows fewer experienced males, this should counteract a relationship between the years played on top level as expertise-related factor and relative UBHS values. Nevertheless, results indicate that the number of years played at the top level was a significant predictor for UBHS. Thus, expert GSA seem to show higher relative UBHS values, which strengthens the expertise-related relevance of our measurement. This, additionally, might be moderated by sport-specificity. In the female sample, ice-hockey players were overrepresented with *n* = 19 compared to other sports. At the same time, female ice-hockey players showed highest UBHS values compared to other female as well as to male GSA. This might be due to the requirements of ice-hockey (e.g., mostly unilateral actions and lateral-shifted positions during passes and shots). In addition, ice-hockey players played the longest at the top level (10.53 ± 5.21) compared to others (4.66 ± 4.37). These findings are in accordance with previous studies by Gonçalves et al. (6) and Milić et al. (7), who found superior upper-body performance for elite compared to sub-elite athletes. However, according to Cohen (31) the *R^2^* for the overall model in the present study is only small. Since we only measured professional athletes in our sample, the small *R^2^* value may be due to a fairly homogeneous group in terms of expertise (number of years played at the top level = 4.35 ± 4.80 years). Similar results were shown for position 3, although not for UBHS in position 1. Therefore, the measurement does not seem to differentiate between the higher and lower expertise athletes in an upright position. Interestingly, this was in accordance with some athletes' statement that this upright position was rather unusual and the least “sport-specific” compared to the other two positions. This may be explained by the setting of the position, in which the athletes had to stand very upright (shoulder over hip joint). Since GSA typically perform game-actions in a leaned forward position (e.g., in handball, the pivot player always uses his whole body to push against his defenders), this position may be the least game-specific. In accordance with this, GSA reported that they found it difficult to push horizontally in this upright position to develop UBHS. This is in line with the UBHS values (see Figures 4, 5). Nevertheless, positions 2 and 3 seem to be valid positions for measuring the UBHS. Both analyses show that the test is a valid indicator for upper-body strength.

The ANOVA results showed a significant interaction effect between position and shift, indicating that strength values are affected differently by positions. For all positions, male and female GSA generated higher strength values when in the even weight distribution condition (50/50%). However, female GSA, when in a clearly leaned forward position (position 3), showed similar strength values when their weight was shifted to the left leg as well as when it was 50/50% distributed. Regarding positions, we found a large main effect for the factor “position” (partial *η*^2^ = 0.86 for the male, partial *η*^2^ = 0.87 for the female GSA). Both male and female GSA showed higher strength values when pushing in the clearly leaned forward positions than in the slightly leaned forward or upright position. Since the body weight plays a substantial role in UBHS, especially in the leaned forward positions, this observation could be explained due to biomechanics. Consequently, we would expect highest values to occur in the clearly leaned forward position (position 3), which is in agreement to our data. We used those outcomes as a manipulation check. Regarding weight-shifted positions, male and female GSA do show a significant difference between the 50/50% and laterally shifted positions, displaying highest strength values when pushing out of an even distribution. Female GSA did not show significant differences between strength values on the left or right sides; however, they did show similar values when shifted to the left during pushing in a clearly leaned forward position. Interestingly, male GSA did show a significant difference between a left and right shift during pushing, with higher strength values when weight was shifted to the left compared to the right side. However, the effect size was rather small and should, therefore, not be over-interpreted. Nevertheless, when looking at the players individually, some players showed deviating values, e.g., with large DIFF between the left and the right side. These DIFF is detected in our setup and could then be addressed in individualized practice. Thus, our setup measuring UBHS cannot only be used for an individual diagnostic of GSA but also for the long- and short-term evaluation of performance level. Moreover, UBHS values can be used to derive both individual strength training exercises and sport-specific practice.

## 5. Study limitations

Given the aim of this study to validate a tool for measuring UBHS in a game-like standing position the study shows some limitations. In this study we focused on the cross-validation between UBHS and GS measurement as a general marker for overall muscle strength. The linear regression between GS and UBHS showed positive indicators for the validity of our setup. However, we did not examine the association with a “gold-standard” test that specifically captures UBHS (e.g., isometric bench press), which should be investigated in future studies. Also, since only professional athletes took part in the project and the times for diagnostics were limited, there was no opportunity to investigate the intra-day reliability or test-retest reliability with a greater number of athletes. Yet, the within-test and test-retest reliability show positive values in terms of the reliability of the measurement.

## 6. Conclusion

The aim of this study was to verify validity and reliability of a whole-body setup for measuring UBHS in a game-like standing position. For reliability, results indicated that all nine positions measured within this setup show excellent levels of intra-class correlation as well as high levels of test-retest reliability of position 2. For validity, linear regression analysis showed that GS, as an indicator of overall muscle strength, significantly predicts UBHS in female but not in male GSA which may be in part attributed to sports specificity. In addition, as an expertise-related factor, UBHS can be predicted by the number of years played at the top level and therefore seems to be a performance-related parameter. Male and female GSA showed highest values when standing with their weight evenly distributed (50/50%) compared to when their weight was shifted onto the left or right leg during pushing. Thus, these results are the first positive indicators that the whole-body oriented setup used in this study might be a valid and reliable method for measuring UBHS. The setup could be beneficial for assessing sport-specific UBHS more precisely, e.g., addressing game-like weight-shifted positions. In future studies, the cross-validation and test-retest reliability should be further investigated. Therefore, we suggest that only position 2, the slightly leaned forward position, should be measured since this position reflects sport specificity (e.g., when setting blocks as a pivot player in handball) most accurately.

## Data Availability

The raw data supporting the conclusions of this article will be made available by the authors, without undue reservation.
